# The Mediating Role of Psychological Resilience in the Relationship Between Presenteeism and Perioperative Competence in Operating Room Nurses

**DOI:** 10.3390/healthcare14213570

**Published:** 2026-10-22

**Authors:** Ayşe Uçak, Arzu Tat Çatal, Semanur Karadeniz, İbrahim Hakkı Tezci

**Affiliations:** 1 Department of Nursing, Faculty of Health Sciences, Burdur Mehmet Akif Ersoy University, Burdur 15100, Türkiye; ayseucak32@gmail.com; 2 Department of Surgical Nursing, Faculty of Nursing, Akdeniz University, Antalya 07058, Türkiye; semanurkrdeniz@gmail.com; 3 Department of Measurement and Evaluation in Education, Faculty of Education, Akdeniz University, Antalya 07058, Türkiye; ihtezci@akdeniz.edu.tr

**Keywords:** operating room nurse, presenteeism, psychological resilience, perioperative competence, mediation analysis

## Abstract

**Highlights:**

**What are the main findings?**

• Presenteeism was negatively associated with both psychological resilience and perceived perioperative competence among operating room nurses.
• Psychological resilience statistically accounted for a modest but significant portion of the association between presenteeism and perioperative competence (31.1% of the total effect). A sensitivity analysis accounting for measurement error yielded a similar pattern of findings.

**What are the implications of the main findings?**

• Presenteeism may merit consideration as an organizational and patient-safety concern, rather than solely as an individual productivity issue.
• Multilevel strategies, including supportive sick-leave policies, appropriate staffing and workload management, and resilience-enhancing programs, may merit evaluation as potential approaches for supporting perioperative competence among operating room nurses.

**Abstract:**

Purpose: This study examined the associations among presenteeism, psychological resilience, and perioperative competence in operating room nurses and whether psychological resilience statistically accounted for part of the association between presenteeism and perioperative competence. Methods: This descriptive, cross-sectional, correlational study included 251 operating room nurses working in public and university hospitals in Türkiye. Data were collected using the Descriptive Information Form, the Perceived Perioperative Competence Scale-Revised, the four-item Turkish short form of the Stanford Presenteeism Scale, and the Brief Resilience Scale. Pearson correlation and mediation analyses were con-ducted using Hayes’ PROCESS macro, adjusting for age, gender, hospital type, professional experience, and shift type. An errors-in-variables sensitivity analysis was also performed. Results: Presenteeism was negatively associated with psychological resilience and perioperative competence, whereas psychological resilience was positively associated with perioperative competence (all *p* < 0.01). The indirect effect through psychological resilience was significant (ab = −0.410, 95% BC CI [−0.828, −0.123]) and accounted for 31.1% of the total effect. The direct effect did not reach statistical significance (c′ = −0.908, *p* = 0.066). The sensitivity analysis showed a similar pattern. Conclusions: Psychological resilience accounted for a modest but significant portion of the association between presenteeism and perioperative competence. Longitudinal studies are needed to clarify the temporal ordering of these relationships.

## 1. Introduction

The operating room is among the most complex clinical environments in healthcare because of its high level of risk, low tolerance for error, need for rapid decision-making [1,2,3], and reliance on multidisciplinary teamwork [4]. Nurses working in this environment require advanced clinical knowledge and technical skills as well as professional perioperative competence [5]. Perioperative competence is a multidimensional construct encompassing surgical field-specific knowledge and technical skills, as well as clinical decision-making, effective communication, and team coordination; it also involves ensuring patient safety, assuming professional responsibility, and providing safe care under stressful conditions [6,7,8]. As a key determinant of high-quality perioperative care, this competence is closely as-sociated with patient safety and the quality of surgical care [9]. Operating room nursing is a complex specialty that requires the integration of cognitive, psychomotor, affective, social, and contextual competencies [1,4,9,10]. At the same time, heavy workloads, time pressure, long working hours, and conditions requiring continuous attention can place considerable strain on nurses’ physical and psychological resources [11,12].

One issue receiving increasing attention among healthcare professionals is presenteeism [13]. Presenteeism refers to continuing to work despite illness, fatigue, pain, psychological distress, or burnout, often accompanied by reduced work performance [14]. A meta-analysis estimated the prevalence of presenteeism among nurses at 49.2%, with estimates ranging from 32% to 94% across individual studies. That meta-analysis also identified high workload, staff shortages, and emotional stress as factors associated with presenteeism [13].

Operating room nurses have critical responsibilities for ensuring that the surgical process is carried out safely, consistently, and effectively in a highly technological and specialized environment [5]. Fulfilling these responsibilities requires not only technical knowledge and skills but al-so sustained cognitive attention, emotional balance, and psychological resilience [1,15,16,17,18]. The association between presenteeism and professional functioning may therefore be especially relevant among operating room nurses. Studies of operating room nurses have linked burnout, emotional exhaustion, and work-related stress with poorer professional functioning [11,12]. Previous studies also indicate that psychological resilience and communication competence are associated with lower burnout and better professional functioning [12,17,19].

Psychological resilience refers to an individual’s capacity to adapt to stress, adversity, traumatic experiences, and intense work demands, maintain functioning, and re-cover from negative experiences [20]. In nursing, psychological resilience is viewed as an important personal resource; high-er resilience has been associated with less burnout, fatigue, and work-related stress, as well as greater professional competence, job performance, job satisfaction, and organizational support [15,16,18]. Studies of operating room nurses likewise suggest that higher psychological resilience is associated with lower levels of burnout and depression, stronger communication competence, and better professional functioning [12,17]. These findings suggest that psychological resilience may be an important personal re-source for sustaining professional functioning under challenging working conditions.

Existing evidence on presenteeism, psychological resilience, and professional func-tioning has largely developed along separate lines of inquiry. Research on presenteeism among nurses has largely focused on productivity- and care-related outcomes, including productivity loss [21] and poorer patient-reported experience [22]. An integrative review further identified workload and organizational climate as fac-tors associated with presenteeism [23]. A second line of research has focused primarily on psychological resilience as a personal resource associated with fatigue, burnout, and occupational functioning [24,25]. A third has focused mainly on perioperative competence in relation to professional competencies, education, and clinical development [9,10]. Taken together, these findings suggest that presenteeism, psychological resilience, and perioperative competence may be interrelated; however, these three constructs have rarely been examined together in a single theoretical model.

Indirect evidence also supports examining psychological resilience within presenteeism related pathways. Psychological capital, a broader construct encompassing resilience-related psychological resources, has been identified as an intervening variable in relationships among social support, burnout, and presenteeism among nurses [26,27]. However, evidence remains limited as to whether psychological resilience statistically accounts for part of the association between presenteeism and perioperative competence among operating room nurses. This question may be especially relevant in the operating room, where professional competence requires sustained attention, decision-making, communication, and psychomotor performance under demanding conditions [1,4,9]. Examining these variables together could therefore extend the literature by bringing occupational functioning, personal psychological resources, and specialty-specific competence into a single theoretical framework.

### Theoretical Framework and Hypothesis Development

This study is grounded in Hobfoll’s Conservation of Resources (COR) Theory [28], which provides the conceptual basis for the proposed associations among presenteeism, psychological resilience, and perioperative competence and for the statistical mediation model tested in this study. COR Theory posits that individuals strive to acquire, protect, and maintain valued resources, broadly defined as objects, personal characteristics, conditions, and energies that are valued in themselves or that facilitate the attainment of other valued outcomes [28]. According to the theory, stress occurs when resources are threatened with loss, actually lost, or insufficiently gained following substantial resource investment. A central proposition of COR Theory is that sustained resource expenditure without adequate recovery or replenishment may reduce the resources available for subsequent functioning and increase vulnerability to further resource loss, thereby contributing to a resource loss spiral [28]. This spiral principle provides a theoretical rationale for the proposed ordering in the present statistical model, in which continued resource expenditure under adverse working conditions (presenteeism) is expected to be associated with a lower level of an available personal resource (psychological resilience), which in turn is expected to be associated with a performance-related outcome (perioperative competence).

Within this framework, presenteeism can be conceptualized as a resource-depleting work condition. Continuing to perform professional duties despite illness, fatigue, pain, or psychological distress may require sustained expenditure of physical, cognitive, and emotional resources under circumstances in which these resources are already compromised and opportunities for recovery may be limited [14,28]. In the operating room, where professional performance requires sustained vigilance, rapid decision-making, communication, coordination, and precise psychomotor functioning [1,4,9], such resource strain may be particularly relevant to perioperative competence. Previous evidence linking presenteeism and mental fatigue with poorer care-related outcomes further supports examination of this association [29,30]. Accordingly, COR Theory provides the theoretical rationale for expecting presenteeism to be negatively associated with perioperative competence (H1).

Psychological resilience can be conceptualized within COR Theory as an important personal resource [28]. It supports adaptation, recovery, and continued functioning under stressful or demanding conditions [15,20,31]. Individuals with greater resilience may be better able to preserve or mobilize psychological resources in response to occupational demands, whereas sustained demands associated with working while unwell may be associated with reduced availability of resilience-related resources. This interpretation is consistent with the COR proposition that resource loss or prolonged resource expenditure may constrain the resources available for coping with subsequent demands [28]. Accordingly, presenteeism is expected to be negatively associated with psychological resilience (H2). This theoretical expectation is also supported by evidence linking resilience with lower burnout, fatigue, and work-related stress [15,16,18].

Psychological resilience, in turn, is expected to be positively associated with perioperative competence (H3). Competent perioperative practice requires nurses to maintain cognitive attention, emotional regulation, communication, clinical decision-making, and coordinated psychomotor performance under demanding and high-risk conditions [1,4,9]. As a personal psychological resource, resilience may support the maintenance of these functions when occupational demands are high. Studies involving operating room nurses have similarly associated greater psychological resilience with lower burnout and depression, stronger communication competence, and better professional functioning [11,17], while broader nursing research has linked resilience with occupational functioning and professional competence [24]. Thus, COR Theory provides a resource-based explanation for the expected positive association between psychological resilience and perioperative competence.

Building on these propositions, COR Theory also provides a rationale for examining whether psychological resilience statistically accounts for part of the association between presenteeism and perioperative competence. The resource loss spiral suggests that resource strain in one domain may be accompanied by reductions in resources available for subsequent functioning [28]. Applied to the present statistical model, if presenteeism is associated with lower psychological resilience (H2) and psychological resilience is positively associated with perioperative competence (H3), then part of the overall association between presenteeism and perioperative competence may be statistically accounted for by psychological resilience (H4). Previous studies showing indirect relationships involving psychological resources, including psychological capital, and presenteeism-related occupational outcomes provide additional empirical support for examining such a pathway [26,27]. This indirect pathway is theoretically complementary to the direct association proposed in H1, because COR Theory does not imply that the associations arising from a resource-depleting condition must operate through a single mechanism.

Importantly, the proposed ordering from presenteeism to psychological resilience and subsequently to perioperative competence should not be interpreted as an empirically established temporal or causal process. All variables in the present study were measured concurrently within a cross-sectional survey. Therefore, the proposed model represents a theory-informed statistical mediation model rather than a demonstrated longitudinal mechanism. Cross-sectional mediation analysis can indicate whether the observed pattern of associations is statistically consistent with a theoretically proposed indirect pathway, but it cannot establish temporal precedence or causal direction, rule out reverse or reciprocal relationships, or exclude the possibility that unmeasured variables account for the observed associations [32,33]. For example, lower perioperative competence could itself be associated with greater occupational strain or presenteeism, and reciprocal relationships between psychological resilience and professional functioning are also possible.

Accordingly, guided by Hobfoll’s Conservation of Resources Theory, this study examined the associations among presenteeism, psychological resilience, and perioperative competence in operating room nurses and assessed whether psychological resilience statistically accounted for part of the association between presenteeism and perioperative competence. Given the cross-sectional design, the hypothesized paths were interpreted as theory-informed statistical associations rather than causal relationships. The following hypotheses were tested:

**H1.**  *Presenteeism is negatively associated with perioperative competence.*

**H2.**  *Presenteeism is negatively associated with psychological resilience.*

**H3.**  *Psychological resilience is positively associated with perioperative competence.*

**H4.**  *Psychological resilience statistically accounts for part of the association between presenteeism and perioperative competence.*

## 2. Materials and Methods

### 2.1. Study Design

This study used a descriptive, cross-sectional, correlational design and was reported in accordance with the Strengthening the Reporting of Observational Studies in Epidemiology (STROBE) checklist. The completed STROBE checklist is provided as Supplementary Material (Table S1). Because all study variables were measured concurrently, the analyses were designed to examine theory-informed statistical associations rather than to establish temporal ordering or causal relationships.

### 2.2. Setting and Sample

The study population comprised operating room nurses employed in public and university hospitals in the city centers of Burdur, Isparta, and Antalya in Türkiye’s Western Mediterranean Region. The researchers requested permission from 27 hospitals across the three city centers. The study was conducted in a total of six hospitals that granted institutional permission, including one university hospital and five public hospitals. During the data collection period, 320 operating room nurses were actively employed in these hospitals. Of these, 35 declined to participate due to workload, fatigue, or unwillingness to participate, while 34 could not be reached during the data collection period because they were engaged in surgical procedures or due to their work schedules. The study was completed with 251 operating room nurses, yielding a participation rate of 78.4%. Nurses were recruited from the participating hospitals using convenience sampling.

The sample size was determined by an a priori power analysis using G*Power 3.1 software. Assuming α = 0.05, power (1 − β) = 0.80, and a medium effect size (f^2^ = 0.15) [34], the minimum required sample size was calculated as 138 participants [35]. To allow for potential data loss, the target sample size was increased to 200 nurses, which exceeded the sample size required to detect a medium mediated effect in regression-based mediation models [36]. The final sample comprised 251 operating room nurses from public and university hospitals that granted institutional permission. No data were missing.

Operating room nurses who had worked actively in the operating room for at least six months and volunteered to participate in the study were included. This period was considered the minimum needed for nurses to adapt to the operating room environment and gain sufficient experience in surgical processes. Working outside the operating room, temporarily holding an administrative position, having less than six months of operating room experience, and wishing to withdraw from the study at any time were listed as exclusion criteria.

Among the 251 participants, mean age was 38.64 ± 9.11 years (range, 23–58), mean professional experience was 16.69 ± 10.28 years (range, 1–40), and mean operating room experience was 11.80 ± 9.26 years (range, 1–45). Mean weekly working time was 41.80 ± 6.31 h (min–max: 40–72). Among the participants, 81.3% were female, 83.3% had a bachelor’s degree, 49.8% worked in shifts, and 55.8% worked as both circulating and scrub nurses (Table 1).

### 2.3. Data Collection and Instruments

Data were collected in person between 18 January 2026, and 12 May 2026, after ethical approval and institutional permissions had been obtained. Participants took approximately 15 min to complete the data collection forms. The data collection instruments were the Descriptive Information Form, the Perceived Perioperative Competence Scale-Revised (PPCS-R), the Stanford Presenteeism Scale (SPS; four-item Turkish short form), and the Brief Resilience Scale (BRS).

The Descriptive Information Form collected participants’ sociodemographic (age, gender, marital status, and educational level) and professional (type of institution, working pattern, role in the operating room, duration of professional experience, and duration of operating room experience) characteristics.

Gillespie et al. [8] developed the PPCS-R to assess perioperative competence in operating room nurses. This study used the Turkish version adapted by Sönmez and Ayoğlu [37]. The scale contains 40 items across six subdimensions—foundational knowledge and skills, leadership, collaboration, proficiency, empathy, and professional development—rated on a five-point Likert-type scale from 1 = strongly disagree to 5 = strongly agree. Total scores range from 40 to 200, with higher scores indicating higher perceived perioperative competence. The Turkish version had a reported Cronbach’s α of 0.97 [37]. In this study, internal consistency was α = 0.98.

Koopman et al. [38] originally developed the six-item SPS, which uses a five-point Likert scale (total score range 6–30). This study used the Turkish adaptation by Teoman and Harmancı-Seren [39], in which two items with item-total correlations below 0.40 were removed during the validation process, yielding a four-item, single-factor short form. The version used in this study therefore comprises four items rated on a five-point Likert scale, with total scores ranging from 4 to 20. Higher scores indicate higher levels of presenteeism. The Turkish adaptation had a reported internal consistency of α = 0.76 [39]; in this study, it was α = 0.63. Because this value was below the commonly used threshold, a sensitivity analysis was conducted to assess whether the results were affected by measurement error.

The BRS was developed by Smith et al. [40]. This unidimensional six-item scale is rated on a five-point Likert scale from 1 = strongly disagree to 5 = strongly agree. The total score ranges from 6 to 30, with items 2, 4, and 6 reverse-coded. Higher scores indicate higher psychological resilience. The Turkish version was validated by Doğan [41], who reported a Cronbach’s α of 0.83. In this study, internal consistency was α = 0.79.

### 2.4. Data Analysis

IBM SPSS Statistics 31.0 (IBM Corp., Armonk, NY, USA) was used for descriptive statistics, reliability analyses, and Pearson correlations among the study variables. Although the Shapiro–Wilk tests for the three variables were statistically significant, skewness (−0.41 to 0.07) and kurtosis (−0.29 to 0.32) values remained within ±1, supporting approximate normality [42]. These distributional characteristics were considered acceptable for regression-based analyses in a sample of this size. There was no evidence of multicollinearity; all variance inflation factor (VIF) values were <1.11. Because the data were collected from a single source using self-report measures, common method variance was assessed preliminarily with Harman’s single-factor test. In the unrotated single-factor solution including all scale items, the first factor explained 40.31% of the total variance, below the commonly used 50% threshold [43]. However, given the limited sensitivity of Harman’s single-factor test, this result was treated only as a preliminary diagnostic and does not exclude the possibility of common method bias.

The proposed mediation model was tested using Model 4 of the PROCESS macro for SPSS (version 5.0), developed by Hayes [44]. The indirect effect was evaluated with bias-corrected bootstrap confidence intervals based on 5000 resamples. The model included age, gender, hospital type, total professional experience, and shift type as covariates. An indirect effect was considered statistically significant when its 95% confidence interval excluded zero [44,45]. Because the Cronbach’s α coefficient of the presenteeism scale (α = 0.63) was below the commonly used threshold, a sensitivity analysis was conducted to assess the potential influence of measurement error. For this sensitivity analysis, the errors-in-variables regression method proposed by Hayes et al. [46] was implemented using PROCESS version 5. This procedure accounts for random measurement error in composite scores using the reliability coefficients of the three scales and recalculates the model coefficients accordingly. Statistical significance was defined as α = 0.05.

### 2.5. Ethical Considerations

The study followed the ethical principles of the Declaration of Helsinki, with measures in place to protect participants’ rights, privacy, and confidentiality. Before data collection, ethical approval was obtained from the Burdur Mehmet Akif Ersoy University Non-Interventional Clinical Research Ethics Committee, dated 14 January 2026, with the approval number GO 2025/2456. Institutional permissions were obtained from the relevant institutions, and written informed consent was obtained from all participants. Participants were informed that they could withdraw from the study at any time without consequence.

## 3. Results

### 3.1. Preliminary Analyses and Descriptive Statistics

Mean scores for presenteeism, psychological resilience, and perioperative competence were 11.35 ± 3.53, 21.06 ± 4.44, and 156.66 ± 27.33, respectively (Table 2). Pearson correlations showed that presenteeism was negatively correlated with psychological resilience (r = −0.31, *p* < 0.01) and perioperative competence (r = −0.18, *p* < 0.01), whereas psychological resilience was positively correlated with perioperative competence (r = 0.21, *p* < 0.01).

### 3.2. Mediation Analysis

**Figure 1:**
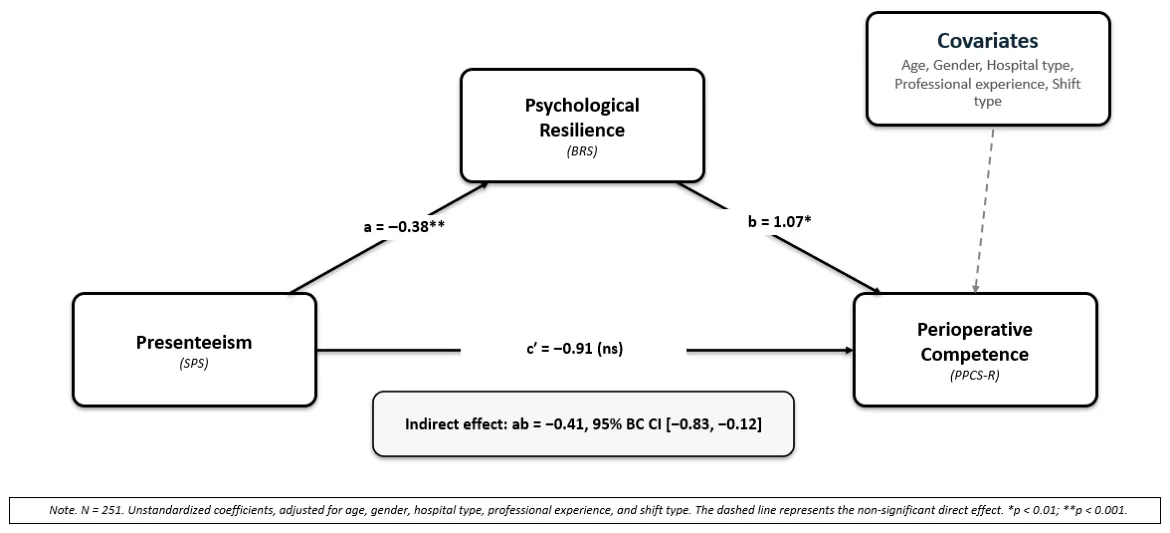
Mediation model of psychological resilience in the association between presenteeism and perioperative competence.

Mediation results are presented in Figure 1 and Table 3.

In the mediation analysis adjusted for age, gender, hospital type, total professional experience, and shift type, presenteeism was negatively associated with psychological resilience (path a: B = −0.382, SE = 0.075, β = −0.30, 95% CI [−0.529, −0.235], *p* < 0.001). Psychological resilience was positively associated with perioperative competence (path b: B = 1.072, SE = 0.401, β = 0.17, 95% CI [0.283, 1.861], *p* = 0.008). The total effect of presenteeism on perioperative competence was negative and statistically significant (path c: B = −1.318, SE = 0.474, β = −0.17, 95% CI [−2.252, −0.384], *p* = 0.006). When psychological resilience was included in the model, the direct effect of presenteeism on perioperative competence did not reach statistical significance (path c′: B = −0.908, SE = 0.493, β = −0.12, 95% CI [−1.878, 0.062], *p* = 0.066).

The indirect effect through psychological resilience was statistically significant (ab = −0.410, BootSE = 0.179, 95% BC CI [−0.828, −0.123]), because the bootstrap confidence interval excluded zero. The indirect effect accounted for 31.1% of the total effect (|ab/c| = 0.410/1.318). Of the covariates, gender (B = 9.76, *p* = 0.029) and hospital type (B = 10.97, *p* = 0.005) were significantly associated with perioperative competence, whereas age, professional experience, and shift type were not. The model accounted for 13.8% of the variance in psychological resilience (R^2^ = 0.138) and 11.5% of the variance in perioperative competence (R^2^ = 0.115).

Because the Cronbach’s α coefficient of the presenteeism scale (α = 0.63) was below the commonly used threshold, a sensitivity analysis was conducted using the errors-in-variables regression method proposed by Hayes et al. [46] to assess the potential influence of measurement error. In this analysis, the reliability coefficients of the scales were incorporated into the same covariate-adjusted model, and the path coefficients were recalculated. In the corrected model, the absolute path coefficients increased (path a: B = −0.609, *p* < 0.001; path b: B = 1.232, *p* = 0.045; total effect: B = −2.100, *p* = 0.003), as did the explained variance (R^2^BRS = 0.193; R^2^PPCS-R = 0.130). The corrected indirect effect involving psychological resilience remained statistically significant (ab = −0.750, 95% BC CI [−1.763, −0.061]). Because the errors-in-variables procedure adjusts estimates based on the reliability coefficients entered into the model, the larger corrected coefficients reflect measurement-error adjustment and should not be interpreted as evidence of substantively stronger associations. Overall, the sensitivity analysis produced a pattern consistent with the primary analysis. However, because the upper limit of the confidence interval was close to zero, this finding should be interpreted cautiously and as supportive, rather than conclusive, evidence of the consistency of the indirect association.

## 4. Discussion

This study examined the relationships among presenteeism, psychological resilience, and perioperative competence in operating room nurses using a theory-informed mediation model. The results indicated that presenteeism was negatively associated with perioperative competence and that psychological resilience statistically accounted for part of this association. Psychological resilience was also positively associated with perioperative competence, suggesting that it may represent a personal resource relevant to professional functioning among operating room nurses. Consistent with Hobfoll’s Conservation of Resources Theory [28], these findings suggest that psychological resources may help sustain professional functioning in high-risk clinical environments.

The first hypothesis (H1) was supported, as presenteeism was negatively associated with perioperative competence among operating room nurses. This finding aligns with previous studies linking presenteeism and poorer clinical performance or care-related outcomes [22,29,30]. Operating rooms are characterized by high risk, time pressure, and the need for continuous team coordination. Accordingly, impaired cognitive or physical functioning may be particularly consequential for the safety and effectiveness of perioperative care [47]. Notably, Rahmani et al. [29] reported that 77.9% of operating room nurses experienced varying levels of mental fatigue, which was significantly and positively associated with disruptions in perioperative care. Similarly, Ji et al. [30] reported that approximately 65% of nurses were present at work but unable to work efficiently, a condition associated with higher rates of missed care.

Perioperative competence includes both technical skills and non-technical competencies, such as clinical decision-making, communication, and teamwork [6,48]. The cognitive and emotional burden associated with presenteeism may compromise performance across both technical and non-technical domains of competence. This interpretation also aligns with the findings of Li et al. [49], who reported that self-efficacy fully mediated the relationship between job burnout and professional performance. Collectively, these findings suggest that psychological and cognitive resources may be important for sustaining professional performance.

Working in surgical settings has been identified as a factor associated with presenteeism [50]. Heavy workloads, unpredictable complications, and working conditions that require continuous attention may contribute to presenteeism and place greater demands on nurses’ physical and cognitive resources [12]. These features may help explain why the association between presenteeism and peri-operative competence is especially important in the operating room setting. However, the mean presenteeism score in the present study (11.35 ± 3.53) was lower than the values reported by Wang and Li [51] (15.94) and Huang et al. [52] (16.13). These differences may reflect variation in sample characteristics, work environments, and measurement instruments.

### 4.1. Relationship Between Presenteeism and Psychological Resilience

The present results indicate that presenteeism is associated with lower psychological resilience among operating room nurses. This result is consistent with previous studies reporting an inverse association between presenteeism and psychological resilience [25,53]. Accordingly, the second hypothesis (H2) was supported. Chiu and Wei [53] reported that higher psychological resilience was associated with lower levels of mental, physical, and emotional fatigue among nurses. Similarly, Yu et al. [25] found that psychological resilience was negatively associated with burnout, psychological problems, and job demands. Because presenteeism involves continuing to work despite health problems, it may coincide with greater physical and psychological strain, which may in turn be relevant to the lower resilience observed among nurses experiencing presenteeism.

In the present study, the negative association between presenteeism and psychological resilience suggests that working despite illness or psychological strain may coincide with fewer available psychological resources. Psychological resilience has been associated with positive thinking, healthy lifestyle habits, and cognitive problem-solving skills, whereas presenteeism may place greater demands on these personal resources and reduce the capacity to cope with stress [31]. This interpretation is also consistent with findings showing that psychological capital is indirectly associated with presenteeism [26] and mediates the relationship between job burnout and presenteeism [27]. Although psychological capital and psychological resilience are distinct constructs, both represent psychological resources that may be relevant to adaptive functioning under demanding work conditions.

Operating room nurses experience substantial psychosocial stress because of heavy workloads, time pressure, and working conditions that require sustained attention [3]. Therefore, the negative association between presenteeism and psychological resilience may have implications not only for nurses’ psychological well-being but also for quality of care and patient safety [21,51,54]. Burnout among operating room nurses has been associated with the interaction of organizational and professional factors [2,55]. Taken together, these findings suggest that presenteeism may be an occupational factor linked to lower psychological resilience among operating room nurses.

### 4.2. Relationship Between Psychological Resilience and Perioperative Competence

In the present study, psychological resilience was significantly and positively associated with perioperative competence among operating room nurses. This finding supports the third hypothesis (H3) and is consistent with previous studies reporting positive associations between psychological resilience and nurses’ professional functioning, clinical performance, and quality of care [1,15,56]. Nurses with higher psychological resilience have been reported to show stronger emotional regulation skills, a greater ability to maintain work capacity under stressful conditions, and more effective recovery of professional functioning after critical incidents [1]. In addition, higher psychological resilience has been associated with greater compassion satisfaction and lower levels of burnout and compassion fatigue [1,56]. Taken together, these findings suggest that psychological resilience may be an important personal resource for psychological well-being and for the professional functioning that underlies perioperative competence.

The positive association observed between psychological resilience and perioperative competence suggests that resilience may be a relevant personal resource supporting the professional functioning of operating room nurses [6,48]. Higher psychological resilience may also support more effective use of non-technical competencies required in the operating room, including sustained attention and rapid decision-making. This interpretation aligns with previous studies reporting positive associations among psychological resilience, clinical performance, and quality of care [1,15,56]. Psychological resilience has been described as a multidimensional construct associated with both individual characteristics and organizational support, educational opportunities, and the work environment [15]. Given the workload, time pressure, and high-responsibility conditions in the operating room [2,3], strategies to strengthen psychological resilience merit further evaluation as a potential way to support perioperative competence.

### 4.3. Mediating Role of Psychological Resilience

A key finding was the statistically significant indirect association involving psycho-logical resilience (ab = −0.410, 95% BC CI [−0.828, −0.123]). Although mediation models involving psychological resources are increasingly used in nursing research, the present study extends this literature by examining associations among presenteeism, psychological resilience, and perioperative competence in operating room nurses. The significant indirect effect suggests that psychological resilience statistically accounted for part of the association between presenteeism and perioperative competence, supporting the fourth hypothesis (H4). Although the indirect effect was statistically significant, the direct effect (c′ = −0.908, *p* = 0.066) did not reach statistical significance, and the indirect pathway accounted for 31.1% of the total effect. Thus, the indirect path-way represented a modest share of the overall association. Accordingly, this pattern should not be interpreted as evidence of strict “indirect-only mediation” as defined by Zhao et al. [57] Rather, the direct association remained non-negligible in magnitude despite not reaching statistical significance and therefore should not be dismissed. Overall, the findings suggest that psychological resilience statistically accounts for part, but not all, of the association between presenteeism and perioperative competence.

Hobfoll’s Conservation of Resources Theory offers a useful framework for interpreting this indirect association [28]. According to the theory, individuals may adapt more effectively to stressful working conditions when valued psychological and other personal resources are preserved, whereas resource loss is associated with greater strain and impaired functioning. Continuing to work despite health problems, fatigue, or psychological strain may require the continued use of already limited resources. Within this framework, psychological resilience may be a personal resource associated with maintaining professional functioning under demanding conditions. The present findings are consistent with this perspective; however, the cross-sectional design precludes conclusions regarding temporal ordering, including whether presenteeism precedes lower psychological resilience or whether lower psychological resilience is associated with subsequent differences in perioperative competence.

The literature suggests that stronger psychological resources are associated with more effective coping with occupational stress and better maintenance of professional functioning among nurses [12]. Similarly, professional identity and a growth mindset have been linked to greater psychological resilience and better adaptation to challenging working conditions [18]. Psychological resilience has also been described as a dynamic construct associated with both individual and contextual factors, including organizational support, educational opportunities, and the work environment [56]. In the present study, psychological resilience accounted for a statistically significant but modest share of the association between presenteeism and perioperative competence. Although the explained variance was modest (R^2^ = 0.115 for perioperative competence; R^2^ = 0.138 for psychological resilience) and the standardized indirect effect was small (β = −0.05), the findings suggest that psychological resilience may be one relevant personal resource associated with perioperative competence, alongside other individual, professional, and organizational factors not captured by the present model.

Taken together, the findings suggest that the association between presenteeism and perioperative competence includes a smaller indirect component through psycho-logical resilience. Perioperative competence appears to reflect not only professional knowledge and technical skills but also psychological resources relevant to functioning in demanding clinical environments. However, given the modest proportion of variance explained by the model, psychological resilience should be regarded as one of several potentially relevant factors rather than as a decisive determinant of perioperative competence. This interpretation is consistent with Hobfoll’s Conservation of Resources Theory, which emphasizes the relevance of personal resources for functioning under conditions characterized by heavy workload, time pressure, and sustained attention requirements [28]. The statistically significant indirect association further suggests that psychological resilience may statistically account for part of the association between presenteeism and perioperative competence, although the temporal and causal ordering of these variables cannot be established from the present cross-sectional data. Accordingly, presenteeism may merit consideration not only in relation to employee health and work productivity but also as a factor associated with perioperative care processes and patient safety [47,48].

### 4.4. Clinical and Organizational Implications

The findings of this study suggest that presenteeism may have implications beyond individual health and may also represent an organizational concern associated with perioperative competence among operating room nurses. Interventions to reduce presenteeism may therefore merit consideration as part of institutional efforts to support employee health, quality of care, and patient safety. However, given the modest effect sizes observed in this study, the effectiveness of such interventions should be tested empirically rather than assumed. Previous studies suggest that flexible work arrangements, non-punitive sick leave policies, psychological counseling services, and stress-management programs may be useful components of efforts to reduce presenteeism [21,51,54].

The observed indirect association involving psychological resilience suggests that resilience may be relevant to the design of potential interventions for operating room nurses. Psychological resilience has been described as a developable characteristic as-sociated with organizational support, communication skills, educational programs, and a positive work environment [11,15,31]. In addition, previous studies have reported that resilience-enhancing interventions have been associated with lower fatigue and better professional functioning among surgical nurses [24].

Maintaining perioperative competence, however, requires more than individual training. Workload management, effective intra-team communication, interprofessional collaboration, and a supportive work environment may support both psycho-logical resilience and perioperative competence [3,47,48]. Accordingly, multilevel organizational strategies may merit consideration as approaches to reducing presenteeism and sustaining safe perioperative care [58]. Because this cross-sectional study did not test an intervention, these implications should be treated as hypotheses for future evaluation rather than as established recommendations.

### 4.5. Strengths and Limitations

A strength of this study is its theory-informed examination of the associations among presenteeism, psychological resilience, and perioperative competence in operating room nurses. Examining the indirect association involving psychological resilience helps assess whether psychological resilience statistically accounts for part of the association between presenteeism and perioperative competence. In addition, using Hobfoll’s Conservation of Resources Theory as the theoretical foundation allowed the findings to be interpreted within a conceptual framework rather than solely in terms of statistical relationships among variables. Conducting the study in public and university hospitals across three provinces introduced some variation into the sample; however, given the single-region, non-probability design, this should not be interpreted as substantially improving the generalizability of the findings. Furthermore, the sensitivity analysis assessing the potential influence of measurement error yielded a similar pattern of findings, providing additional support for the robustness of the observed pattern of associations.

However, this study has several limitations. First, the cross-sectional design does not allow the temporal ordering specified in the proposed mediation model to be empirically established, thereby limiting causal interpretation of the relationships among the study variables. Because presenteeism, psychological resilience, and perioperative competence were measured concurrently, the estimated indirect effect should be interpreted as a theory-informed statistical association rather than as evidence of a temporal or causal mediating process. Indirect-effect estimates derived from cross-sectional data may differ from the underlying longitudinal mediation process and may therefore be biased when interpreted as evidence of mediation over time [32,33]. In addition, the directional model tested in this study does not rule out reverse or reciprocal relationships. For example, lower perioperative competence may increase work-related strain and, in turn, be associated with higher presenteeism. Longitudinal studies with repeated measurements and, where appropriate, experimental designs are needed to establish temporal ordering and clarify the direction of these relationships.

Second, the use of self-report measures may increase the risk of social desirability and common method bias. Although Harman’s single-factor test indicated that no single factor accounted for a dominant proportion of the variance, it has limited sensitivity in detecting common method variance. More robust approaches, such as including a marker variable or a latent common method factor, were not used. Therefore, given the single-source, self-report design, common method bias cannot be excluded.

Third, the internal consistency of the presenteeism scale in the present sample (α = 0.63) was lower than that reported in the Turkish adaptation study (α = 0.76). This difference may reflect the small number of items in the short form, which makes coefficient alpha more sensitive to sample-specific response patterns, as well as characteristics of the operating room context that may influence item functioning. An errors-in-variables sensitivity analysis was conducted to evaluate the potential influence of measurement error; however, its findings should be interpreted cautiously.

Fourth, the use of non-probability sampling within a single geographical region may limit the generalizability of the findings to operating room nurses working in other settings or regions.

Finally, although psychological resilience was examined as the theoretically proposed variable that may statistically account for part of the association between presenteeism and perioperative competence, other potentially relevant factors, including workload, shift work, burnout, organizational support, social support, and self-efficacy, were not included in the model. Future studies incorporating these variables may provide a more comprehensive understanding of the factors associated with presenteeism and perioperative competence among operating room nurses.

### 4.6. Implications for Research

Future research should examine relationships among presenteeism, psychological resilience, and perioperative competence using longitudinal and, where feasible, experi-mental designs. Incorporating variables such as workload, burnout, organizational support, and shift patterns may provide a more comprehensive account of the pathways linking presenteeism, psychological resilience, and perioperative competence. The effects of interventions aimed at strengthening psychological resilience on perioperative competence should also be evaluated in appropriately designed longitudinal or experimental research.

### 4.7. Implications for Practice

The findings suggest that identifying presenteeism early and considering psychological resilience may be relevant to the management of operating room nurses. Nurse managers may consider organizational strategies to manage workload and working hours, discourage working while ill, and foster a supportive work environment. Resilience enhancing programs may also merit consideration as part of continuing professional development, although their contribution to perioperative competence requires evaluation in future intervention studies.

## 5. Conclusions

This study found that presenteeism was negatively associated with perioperative competence among operating room nurses, whereas psychological resilience statistically accounted for part of this association. The findings suggest that presenteeism may be relevant not only to individual productivity loss but also to lower perceived perioperative competence. Psychological resilience was positively associated with perioperative competence and may represent one personal resource that supports professional functioning in the operating room.

These findings suggest that individual and organizational strategies to reduce presenteeism and strengthen psychological resilience may merit consideration in efforts to support perioperative competence among operating room nurses. Safe staffing, appropriate workload management, a supportive work environment, and resilience enhancing programs may be useful components of such efforts. Future longitudinal and experimental studies examining these relationships across diverse clinical settings are needed to provide stronger evidence regarding the roles of presenteeism and psychological resilience in perioperative competence.

## Figures and Tables

**Table 1:** Participant Characteristics (N = 251).

Variables		n	%
Gender	Female	204	81.3
	Male	47	18.7
Marital status	Married	166	66.1
	Single	82	32.7
	I don’t want to specify	3	1.2
Educational Status	High School/Associate Degree	16	6.4
	Undergraduate	209	83.3
	Postgraduate	26	10.4
Institution	Public hospital	161	64.1
	University hospital	90	35.9
Working Method	08–16	87	34.7
	24 h shift	39	15.5
	Mixed Shift work	125	49.8
Perioperative Nursing Role	Scrub nurse	99	39.4
	Circulation and scrub nurse	140	55.8
	Responsible operating room nurse	12	4.8

**Table 2:** Descriptive statistics, internal consistency coefficients, and Pearson correlation analysis results for the research variables.

Variables	$\overset{-}{X}$	SD	α	1	2	3
Presenteeism ^1^	11.35	3.53	0.63	-		
Psychological resilience ^2^	21.06	4.44	0.79	−0.31 **	-	
Perioperative competence ^3^	156.66	27.33	0.98	−0.18 **	0.21 **	-

Note. N = 251. ^1^ = Presenteeism, ^2^ = Psychological resilience, ^3^ = Perioperative competence. $\overset{-}{X}$ = Mean, SD = Standard deviation, α = Cronbach’s alpha coefficient. Pearson correlation coefficients are reported. ** *p* < 0.01. –: Not applicable.

**Table 3:** Regression coefficients for the mediating analysis of psychological resilience in the effect of presenteeism on perioperative competence.

Effect		B	SE	β	%95 CI
Direct Effect	SPS → BRS (a path)	−0.382 ***	0.075	−0.30	[−0.529, −0.235]
	BRS → PPCS-R (path b)	1.072 **	0.401	0.17	[0.283, 1.861]
	SPS → PPCS-R (c′ path)	−0.908	0.493	−0.12	[−1.878, 0.062]
Total Effect	SPS → PPCS-R (c path)	−1.318 **	0.474	−0.17	[−2.252, −0.384]
Indirect Effect (5000 BC bootstrap)	SPS → BRS → PPCS-R (ab path)	−0.410 *	0.179	−0.05	[−0.828, −0.123]

Note: N = 251. SPS = Stanford Presenteeism Scale (four-item Turkish short form); BRS = Brief Resilience Scale; PPCS-R = Perceived Perioperative Competence Scale-Revised; B = Unstandardized coefficient; β = Standardized coefficient; CI = Confidence interval; BC = Bias-corrected bootstrap confidence interval. Bootstrap confidence interval reported with 5000 resamplings for indirect effects. * *p* < 0.05; ** *p* < 0.01; *** *p* < 0.001.

## Data Availability

The data presented in this study are available from the corresponding author upon reasonable request. The data are not publicly available due to privacy and ethical considerations.

## Supplementary Materials

The following supporting information can be downloaded at: https://www.mdpi.com/article/10.3390/healthcare14213570/s1, Table S1: STROBE Statement—Checklist of items that should be included in reports of cross-sectional studies.

## Abbreviations

The following abbreviations are used in this manuscript:

COR	Conservation of Resources Theory
SPS	Stanford Presenteeism Scale (four-item Turkish short form)
BRS	Brief Resilience Scale
PPCS-R	Perceived Perioperative Competence Scale–Revised
